# Validity of the use of a subfascial vessel as the recipient vessel in a second free flap transfer: A retrospective clinical review

**DOI:** 10.1097/MD.0000000000009819

**Published:** 2018-02-02

**Authors:** Sang Won Seo, Kyu Nam Kim, Won Ha, Chi Sun Yoon

**Affiliations:** a Department of Emergency Medicine, Eulji University Hospital, University of Eulji College of Medicine, Seo-Gu, Daejeon; b Department of Plastic and Reconstructive Surgery, Konyang University Hospital, University of Konyang College of Medicine, Myunggok Medical Research Center, Daejeon; c Department of Plastic and Reconstructive Surgery, Ulsan University Hospital, University of Ulsan College of Medicine, Dong-Gu, Ulsan, Korea.

**Keywords:** perforator, recipient vessel, second free flap, supermicrosurgery

## Abstract

Performing a greater number of free flap procedures inevitably results in an increase in the number of cases that experience free flap failure. In cases that require a second free flap after the failure of the first, recipient vessel selection becomes difficult. Furthermore, recipient vessel selection can be complicated if the vessel is deep in the recipient site, or if there is an increased risk of vessel damage during the dissection. Thus, we present our experience where a subfascial vessel beneath the deep fascia was used as a recipient vessel for a second free flap in lower extremity reconstruction due to total or partial first flap failure.

Between January 2010 and April 2015, 5 patients underwent second free flap reconstruction using a subfascial vessel as the recipient vessel. The flaps were anastomosed in a perforator-to-perforator manner, using the supermicrosurgery technique. We measured the sizes of the flaps, which varied from 5 × 3 to 15 × 8 cm, and the recipient subfascial vessel diameters.

The mean time for the dissection of the recipient perforator was 45 minutes. All the flaps exhibited full survival, although a partial loss of the skin graft at the flap donor site was observed in 1 patient; this defect healed with conservative management.

We recommend using a subfascial vessel as the recipient vessel for both first and second free flaps, especially if access to the major vessel is risky or challenging.

## 1 Introduction

The use of free flaps is a standard technique for lower extremity defect reconstruction because the lower extremities do not have sufficient surrounding tissue to create a local flap (unlike other parts of the body) and are likely to sustain injuries that may restrict the use of local flaps.^[1]^ There are numerous reports regarding the selection of an appropriate donor flap and recipient vessel and the various vascular anastomosis methods that can be used when performing a free flap procedure.^[1–7]^ However, the reported total free flap failure rate is 4% to 10%,^[8–11]^ and partial flap failure is also occasionally observed.^[12]^ Therefore, reconstructive microsurgeons encounter cases of flap failure after lower extremity reconstruction with a free flap. Furthermore, in a situation where a second free flap is required (due to total or partial flap failure), the resulting defect can be worse than the original defect, which makes the selection of the recipient vessel difficult during surgery planning. Therefore, flap failure can affect both the patient and the decisions made by the surgeon.

There are several types of workhorse flaps, such as the anterolateral thigh (ALT) perforator and thoracodorsal artery perforator flap. These well-known flaps provide options for the selection of the donor site. In contrast, the number of suitable recipient vessels is limited. Therefore, we report our experience where a reliable subfascial vessel was used as a recipient vessel in a second free flap procedure through the application of the supermicrosurgery concept. This technique may help expand the limited number of recipient vessels that can be used for second free flaps during lower extremity reconstruction.

## 2 Methods

### 2.1 Ethical approval

All patients provided their informed consent before undergoing the surgical procedure. The study's design was reviewed and approved by the institutional review board of Ulsan University Hospital (approval number: UUH 2015-03-049).

### 2.2 Patients

Between January 2010 and April 2015, 5 patients received a second free flap using a perforator as the recipient vessel, at our institution. All the procedures were performed by the corresponding author (CSY). The defects were located in the knee in 3 patients and in the proximal lower leg in 2 patients and were caused by trauma in 3 patients and chronic osteomyelitis in 2 patients.

### 2.3 Interventions

The mean period between the first and second free flap procedures was 6 weeks (range, 4–8 weeks). For the first free flap, a major vessel was used as the recipient vessel in 1 patient and a perforator was used in 4 patients. After the first free flap procedure, total flap failure occurred in 1 patient and partial flap failure in the other 4 patients. For the second free flap, we used the ALT flap in 1 patient, the superficial circumflex iliac artery perforator flap (SCIP) in 2 patients, and the peroneal artery perforator flap (PAP) in 2 patients. The flap size and the diameter of the recipient subfascial vessel were measured (Table 1).

**Table 1:**
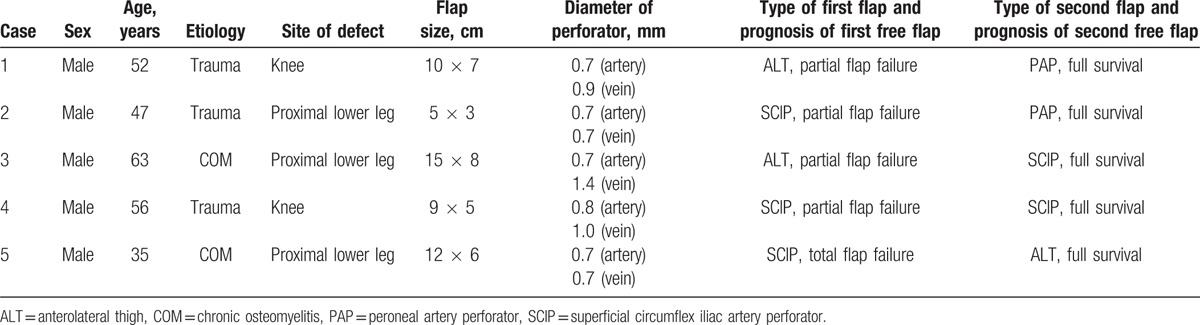
Patient and flap characteristics.

#### 2.3.1 Surgical technique

Vascular status was assessed using computed tomography angiography data between the 2 free flap procedures. Using a handheld Doppler, the strength and location of the signal were marked around the defect, while avoiding the dissection site for the recipient vessel of the first free flap. After performing complete debridement at the first free flap site, we identified and dissected the recipient subfascial vessel for the second free flap under loupe magnification. Superficial veins were preserved during the identification of the perforator. A microscope was used during the dissection of the perforator to minimize damages to the recipient subfascial vessel. After the dissection of the subfascial vessel, the adequacy of blood flow was confirmed by transecting the artery; if the blood flow was not reliable, another subfascial vessel was used. After preparing the recipient subfascial vessel, the PAP, SCIP, or ALT flap was designed and elevated based on the volume of the defect. An appropriate pedicle length was achieved by allowing a generous margin (beyond the required pedicle length). After moving the surgical field to the recipient site, the flap was anastomosed to the defect using the end-to-end method with a nylon 10-0 suture, and a nylon 9-0 suture was used for the anastomosis to a superficial vein. We used a demagnetizer to remove static electricity, and multiple pieces of cotton were placed beneath the site to enable easy anastomosis at a relatively superficial position. After confirming that there was no leakage after the anastomosis, the flap was inserted while avoiding the compression of the pedicle.

## 3 Results

### 3.1 Clinical characteristics

The flap sizes ranged from 5 × 3 to 15 × 8 cm. The ALT and SCIP flaps were both elevated within the superficial fascial plane, and the PAP flap was elevated within the deep fascial plane. Because we used a recipient subfascial vessel beneath the deep fascia for easy vessel anastomosis, the diameter of the recipient subfascial vessel was >0.7 mm for arteries and veins. Preparation of the recipient subfascial vessel required an average duration of 45 minutes (range, 30–70 minutes). However, a longer-than-expected preparation time was required when the injury zone around the defect was large or when the injury zone had expanded due to recipient vessel dissection during the first free flap procedure. All anastomoses were performed in an end-to-end fashion and a superficial vein was used as the recipient vein in 1 patient. All the second flaps fully survived, although 1 patient experienced partial skin loss at the donor skin graft site. The loss was successfully treated via conservative management (including foam dressing). The study follow-up period ranged from 7 to 48 months except in 1 patient (case 1, described below). Complications related to the aesthetic outcome (scar, flap volume) were not observed during follow-up.

#### 3.1.1 Clinical outcomes for case 1

A 52-year-old Chinese man had a head-on collision with a car while driving a motorcycle, and underwent orthopedic treatment for open right patella fracture, anterior and posterior cruciate ligament injury, and right femur fracture. He was transferred to our hospital for the treatment of a residual soft tissue defect in his right knee. The fractures, which involved the right lower extremity, and the blunt soft tissue injury limited the use of a local flap, such as a distally based ALT flap; therefore, a free flap procedure was planned.

**Figure 1:**
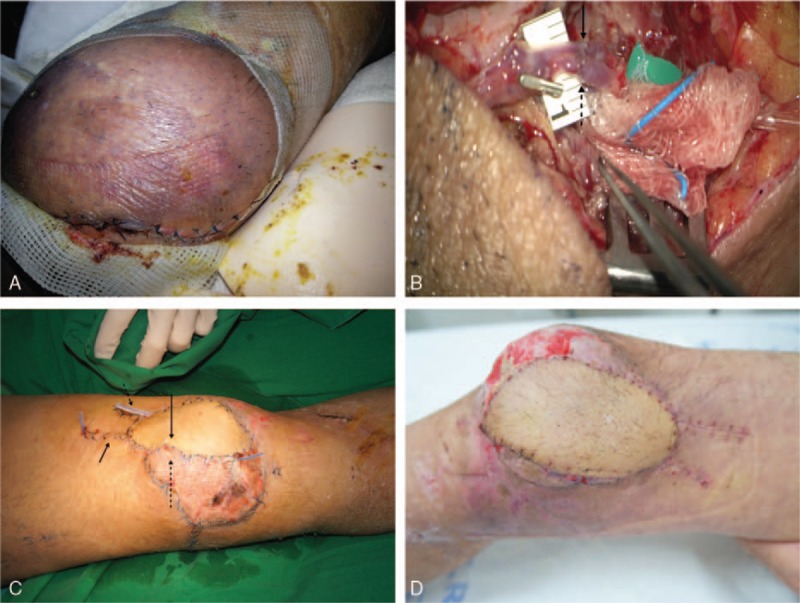
Case 1 (A) Capillary refill is not observed in the flap; flap shows an atypical color change 8 days after the surgery. (B) After the vascular anastomosis; the solid arrow indicates the arterial anastomosis; the dotted arrow indicates the venous anastomosis. (C) Immediately after the completion of the second soleus and peroneal perforator flap: the long solid arrow indicates the second soleus and peroneal flap, and the short solid arrow indicates the second vascular anastomosis area; the long, dotted arrow indicates the first anterolateral thigh perforator flap, and the short, dotted arrow indicates the first vascular anastomosis area. (D) Three weeks after surgery.

The major vessel was located deep within the knee and thus, was not easily accessible. A perforator was used as a recipient vessel to prevent damage during recipient vessel preparation and vascular anastomosis. A 13 × 7.5 cm ALT free flap was designed. The postoperative outcome was good, and there was no arterial insufficiency or venous congestion. However, the patient experienced a fall 7 days later, after which the capillary refill in the flap was no longer observed. Flap necrosis eventually developed and we performed debridement after the demarcation of its depth and extent. Because the necrosis developed from within the flap and spread to the full thickness of the flap, a second free flap procedure was planned. We selected one of the many reliable subfascial vessels around the knee as the recipient vessel of the second free flap for the same reason as that in the choosing of a perforator for the first flap procedure. A 10 × 7 cm soleus and peroneal perforator free flap was performed. The postoperative outcome was good, but the patient was lost to follow-up because he returned to China immediately after his discharge from the hospital on the postoperative day 30. The operative procedure is illustrated in Figure 1.

#### 3.1.2 Clinical outcomes for case 2

**Figure 2:**
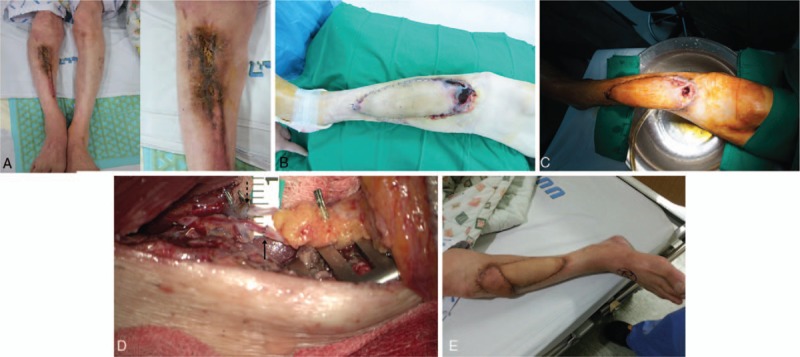
Case 2 (A) Right pretibia osteomyelitis defect. (B) Partial flap failure at the flat upper portion. (C) The bone and anti-beads are exposed. (D) After the vascular anastomosis: the solid arrow indicates the arterial anastomosis; the dotted arrow indicates the venous anastomosis. (E) Two months after the second free flap transfer.

A 63-year-old man with a 10-year history of chronic osteomyelitis involving the right tibia had undergone several surgeries at another hospital, with no improvement, before transferring to our hospital for treatment. His comorbidities included diabetes, heavy smoking, and alcoholism. Curettage and antibead insertion were performed by our orthopedic surgeons. We performed an ALT free flap procedure using the posterior tibial vessel as the recipient, resulting in a 25 × 7 cm flap. After the surgery, partial necrosis developed in the upper part of the flap covering the proximal tibia; we performed debridement after the demarcation of the necrotic depth and extent. Because the bone and antibead were partially exposed, a second free flap procedure was planned. We used a subfascial vessel as the recipient vessel because the defect was in the proximal lower leg where the major vessel was too deep to serve as a recipient. A 15 × 6 cm SCIP free flap was performed. The postoperative outcome and long-term follow-up were good. The operative procedure is illustrated in Figure 2.

## 4 Discussion

Total flap failure can be caused by infection and arterial or venous thrombosis.^[8–11,13]^ Partial flap failure can develop due to the unpredictable characteristics of the perforasome perfusion, as well as other variables that may affect the perfusion of the flap. However, partial failure can still develop even if the aforementioned variables are controlled before surgery.^[12]^ If the first free flap fails, a second free flap is justifiable as the reconstruction goal (defect coverage) has not been achieved.

Various factors must be considered when designing the second free flap (as well as the first free flap); these factors include the choice of the donor flap, the recipient vessel, and the vascular anastomosis method.^[1–7]^ There are several suitable options for the second free flap donor site including the site opposite the failed flap, a site that can provide a longer pedicle length or a muscle flap from the first donor site.^[11]^ However, there is a limited number of suitable recipient vessels for the second flap, and they are more difficult to access. The first vascular anastomosis site becomes the injury zone, forcing it away from the defect during the recipient vessel preparation. Therefore, an interpositional vein graft may be necessary as re-using a major vessel as the recipient vessel for the second free flap may increase the risk of major vessel injury.

The supermicrosurgery concept was introduced by Koshima et al.^[14,15]^ and has since been applied to the field of reconstructive plastic surgery. Numerous reports have demonstrated that the supermicrosurgery approach is effective in cases where the choice of a recipient vessel is complicated by the major vessel being located deep within the target tissue, increasing the risk of major vessel injury during recipient vessel dissection^[16–18]^ or in cases of occlusive vascular disease.^[19]^

**Figure 3:**
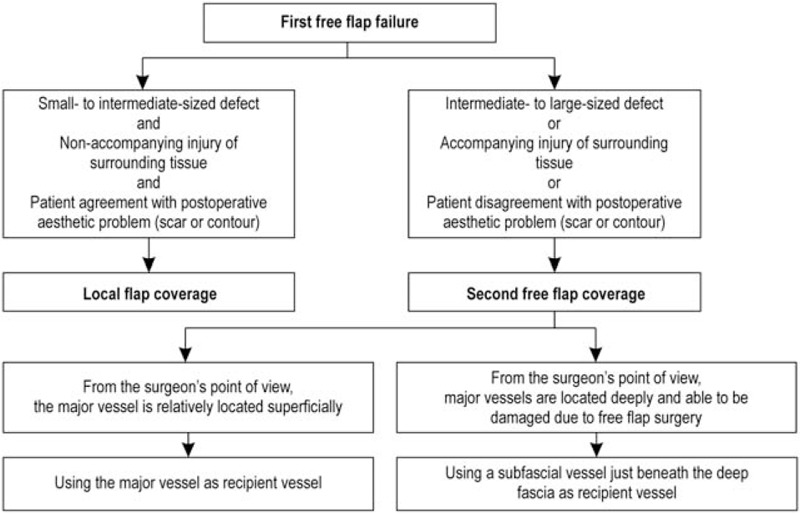
Flowchart for a recipient vessel selection after the failure of the first flap.

In this report, we used the supermicrosurgery concept to perform second free flap procedures using a subfascial vessel as the recipient vessel to reconstruct defects of the knee, proximal lower leg (where the major vessel is located deep in the target tissue), or both. Our institutional algorithm for the recipient vessel selection after the failure of the first flap is illustrated in Figure 3.

Although the use of a perforator as the recipient vessel for the first free flap has previously been reported, the use of a subfascial vessel as the recipient vessel for a second free flap after the failure of the first has not been described. All the second flaps in this series exhibited full survival throughout the follow-up period. In addition, the surgical approach required only an average of 45 minutes for the preparation of the recipient perforator; and we also decreased the flap harvest and pedicle dissection times. Furthermore, the anastomosis was relatively simpler than using a perforator as the recipient vessel, as in the previous study (artery diameter: ranging from 0.4 to 0.9 mm and vein diameter: ranging from 0.4 to 1.2 mm),^[16]^ because the diameter of the recipient subfascial vessel was >0.7 mm for arteries and veins. Moreover, the recipient subfascial vessel was beneath the deep fascia, and the vascular anastomosis was performed in a superficial position (rather than involving a deep major vessel). During the long-term follow-up period, complications involving the scar (instability or undesirable cosmetic outcome) were not observed and functional morbidity related to the flap (in the donor and recipient sites) were not observed.

Our study had some limitations. First, free flap reconstruction may not be the first rung on the reconstruction ladder when considering the standard techniques for the repair of defects involving the region between the thigh and the upper part of the lower leg. A local flap procedure may be suitable for such defects; moreover, designing the flap is more difficult as a reconstruction after the first flap has failed. Additionally, complications such as scarring may develop after the reconstruction of defects above a moderate size. Although our patients had good flap survival without complications, this was a nonrandomized and retrospective study. Furthermore, although preoperative color Doppler ultrasonography imaging was used in a previous study, it was not used for recipient perforator mapping^[20]^; instead, we identified the recipient subfascial vessel using a freestyle approach to enhance cost effectiveness. A well-designed prospective study with a larger sample size is needed to investigate the limitations and drawbacks of our technique. In the future, we plan to compare success rate, operating time, and complications between the use of major vessels and subfascial vessels as recipient vessels.

In conclusion, we believe that a subfascial vessel should be considered as a recipient vessel for a second free flap procedure, as well as for the first, when the surgical approach and dissection are complicated by the location of the major recipient vessel being deep within the target tissue or when there is an increased risk of damage due to repeated recipient vessel dissection. Furthermore, we recommend that the subfascial vessel beneath the deep fascia be used as the recipient vessel, for easy vascular anastomosis.
